# Opinion Piece: Tools for Particle-Size-Based Homogeneity Assessments in Mycotoxin Analysis

**DOI:** 10.3390/foods14193294

**Published:** 2025-09-23

**Authors:** Kai Zhang, Grace Reichard

**Affiliations:** Food and Drug Administration, Human Foods Program, Office of Chemistry and Toxicology, 5001 Campus Drive, College Park, MD 20740, USA; grace.reichard@fda.hhs.gov

**Keywords:** mycotoxin, homogeneity, particle size analysis

## Abstract

Due to the heterogeneous nature of fungal and mycotoxin contamination in foods, it is essential to assess sample homogeneity to ensure that test portions are representative of the entire sample. In mycotoxin analysis, sample homogeneity can be characterized either by the distribution of the target mycotoxin or, more practically, by sample particle size and size distribution. At present, no standardized methodology has been established for assessing sample homogeneity in mycotoxin testing. Moreover, research dedicated to particle-size-based homogeneity assessment, particularly employing particle size analysis tools, remains limited. Therefore, it is worthwhile to discuss available techniques for particle size analysis. This opinion piece presents our perspective on existing techniques, namely sieving, microscopy, laser diffraction, and flow imaging microscopy, while highlighting the current challenges and outlining prospective directions for advancing homogeneity evaluation in mycotoxin analysis.

## 1. Introduction

Mycotoxins are toxic compounds produced by fungi that can contaminate a wide range of food products [1,2,3,4]. Since current agricultural and food processing practices cannot eliminate fungal or mycotoxin contamination from the food supply chain, dietary intake of contaminated food is the primary route of exposure to mycotoxins [3,5]. To protect public health, regulatory authorities have established regulatory limits for mycotoxins—such as aflatoxins, deoxynivalenol, fumonisins, ochratoxin A, and patulin—and monitor their occurrence and concentrations in agricultural commodities and food products via routine mycotoxin analysis; thereby, critical data are generated to support regulatory compliance, dietary exposure studies, health assessments, and risk management [6,7,8].

**Figure 1:**
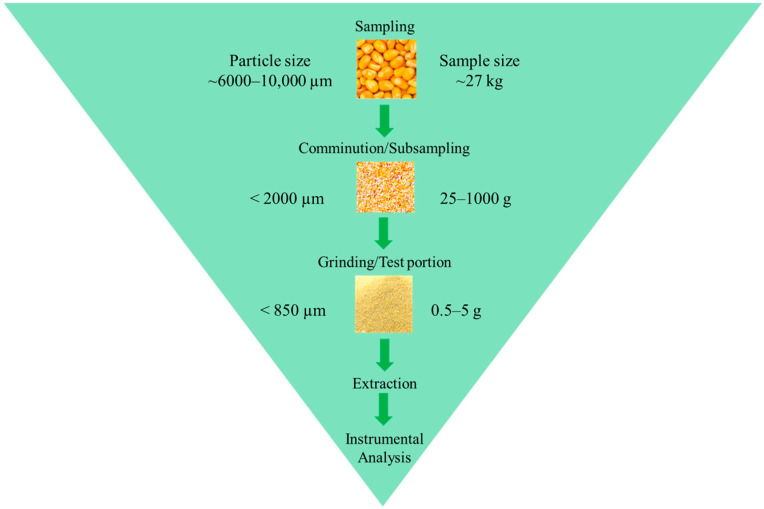
Particle size reduction for mycotoxin sample preparation (given the diversity of mycotoxin sampling procedures, we reference a sample size of 27 kg from the FDA Investigations Operations Manual [10] and the 850 µm particle size threshold as recommended by AOAC 997.16 [15] to illustrate sample size and particle size reduction).

Mycotoxin analysis begins with sample collection from the field, following the establishment of sampling plans [6,9,10,11]. Once collected, these samples are transported to laboratories for both qualitative and quantitative analyses. Figure 1 outlines the primary steps involved in post-collection mycotoxin analysis, highlighting the importance of homogenization in reducing sample particle size and of assessing sample homogeneity to ensure that representative portions can be used for testing. This practice is crucial because fungal growth on crops or food products is rarely uniform—mycotoxins often accumulate in localized “hot spots” with significantly higher toxin concentrations than neighboring areas [12,13,14]. If a sample containing mycotoxins is analyzed without sufficient homogenization, these “hot spots” can cause significant measurement errors (e.g., high false-negative rates or overestimated concentrations) when only a portion of the sample is used for analysis. However, processing the entire sample, which may weigh up to 27.3 kg (60 lbs) [10], is impractical due to the need for large-scale laboratory equipment, high consumable usage (e.g., solvents), and excessive laboratory waste, leading to low sample throughput and high operational costs. 

To minimize the impact of these “hot spots” and improve operational efficiency, primary sampling should consider bulk composite mixing prior to comminution by milling, blending, mixing, and/or shaking, which break the “hot spots” into small particles and promote a more uniform distribution of target mycotoxins. Using particle size analysis, the physical homogeneity of the ground sample can be assessed to verify uniformity prior to test portion selection for extraction and analysis. In this approach, much smaller test portions can be collected and used for analysis, providing representative concentrations of the target mycotoxins without analyzing the entire sample [15,16,17]. 

Furthermore, as advances in analytical technologies have enabled the use of smaller test portions in mycotoxin analysis, the need for assessment of sample homogeneity has become increasingly critical. Conventionally, to compensate for the insufficient sensitivity of techniques like thin-layer chromatography (TLC), large test portions and concentration steps were required to detect aflatoxins at ppb levels [15,18]. Today, advanced liquid chromatography–mass spectrometry (LC–MS) enables “dilute-and-shoot” sample preparation [19,20], significantly reducing both the required test portion size and solvent consumption [21,22]. However, this advancement introduces new challenges: smaller test portions require a higher degree of sample homogeneity to offset increased sampling uncertainty [23,24]. A tenfold increase in analytical sensitivity does not justify a proportional reduction in test portion size without risking additional sampling bias. Thus, when test portion size can be reduced as a result of improved detection limits, it should be complemented by enhanced sample homogenization—or, less ideally, by increasing replication or test portion size—to mitigate the associated sampling error. This strategy is routinely applied in the development of certified matrix reference materials for LC–MS-based mycotoxin analysis [25,26]. In addition to sampling considerations, the physical characteristics of the sample can influence analytical performance. Mycotoxins are often extracted from suspected food samples using extraction solvents, followed by sample clean-up and instrumental analysis. Sample particle size can influence extraction efficiency—the mass transfer rate of target mycotoxin from the food matrix to the solvent—with finer particles offering more surface area per unit mass, thereby enhancing extraction efficiency [27,28,29,30].

Despite its vital role in mycotoxin analysis, mycotoxin sample homogeneity assessment for routine testing remains underexplored, with only a limited number of studies addressing this issue [13,31,32,33,34,35]. ISO Guide 33405 [35] provides a detailed protocol to characterize sample homogeneity, which involves analyzing a large number of test portions (e.g., 30) from a single sample. While effective, this approach is resource-demanding and is therefore considered impractical for routine testing. Alternatively, homogeneity can be inferred from sample particle size and its distribution. 

It is important to note that particle-size-based assessments of homogeneity serve only as an indirect proxy for mycotoxin distribution. A common misconception is that reducing particle size automatically ensures more uniform distribution of mycotoxins. In practice, apparent uniformity in particle size is often mistaken for true homogeneity, yet this perspective fails to consider that mycotoxins may accumulate in specific fractions of the food matrix. Consequently, samples with similar particle size distribution can still have chemical heterogeneity.

Gy’s Sampling Theory provides a quantitative framework that characterizes the relationship between particle size, sample mass, and sampling error [23,24]:

σ^2^ = C·d^3^/m

where

σ^2^ is variance due to fundamental sampling error;

C is Gy’s constant (material-dependent, accounts for heterogeneity);

d^3^ is cube of the particle diameter;

m is mass of the test portion.

This simplified equation implies that halving the particle diameter allows for an eightfold reduction in test portion mass without increasing sampling error (assuming the test portion << the entire sample mass). It highlights the value of particle size analysis as a practical proxy for assessing sample homogeneity. Gy’s Sampling Theory illustrates that reducing particle diameter can significantly decrease sampling error using the same test portion or enable the use of smaller test portions without increasing sampling error. Consequently, the ability to reliably assess particle size and size distribution would significantly improve sampling error control and enhance the understanding of sample homogenization protocols and tools. Given its critical role, the practice of particle size analysis warrants thorough discussion. This opinion piece evaluates four particle size analysis techniques—sieving, microscopy, laser diffraction particle size analysis, and flow imaging analysis—to assess their suitability in mycotoxin sample homogeneity assessments. By comparing their respective strengths and limitations, the goal is to guide the selection of fit-for-purpose homogeneity assessment methods based on particle size that ensure analytical quality and confidence in results. 

## 2. Particle Size Analysis Tools

### 2.1. Sieving

To assess mycotoxin sample homogeneity based on particle size, established standards—such as ISO 16050, EN 14123, and AOAC Official Method 977.16 [15,36,37]—recommend sieving samples using specified particle size thresholds. Sieving involves the physical separation of sample particles based on size, using a single sieve with a pre-defined mesh diameter or a series of sieves with different mesh diameters (e.g., from top to bottom with decreasing diameters). This way, the percentage mass for each size fraction can be measured relative to the starting sample mass and reported as mass-based particle size distribution [29]. The objective is to obtain a more uniformly sized fraction from the entire sample, typically representing the portion suitable for subsequent analytical procedures. Sieving contributes to homogeneity in two principal ways: first, by removing large and inconsistent particles that may increase heterogeneity in subsamples, and second, by facilitating a representative distribution of particles containing mycotoxins across the entire sample via shaking and mixing. This is particularly important in solid or powdered matrices, such as grains, nuts, or animal feed, where mycotoxins may not be evenly distributed due to their localized production by fungal contaminants [38,39]. Additionally, sieves should be cleaned before and after each use to minimize cross-contamination.

Although using a single mesh size for sieving removes coarse particles and enhances the uniformity of the sample matrix, it does not provide detailed particle size distribution data. Such limited information is insufficient for accurately assessing sampling error—unless one assumes a “worst-case scenario” where all particles are considered to be the same size as the mesh opening. To obtain more precise particle size distribution data, multiple sieves would be required, though this method may be cumbersome and time-consuming [40]. 

The mass proportion of the sieved fraction relative to the total sample must be taken into account, as analyzing only the retained portion without such adjustment can introduce bias [41]. When a substantial fraction of the sample (e.g., >50% by mass) is retained on the sieve, the entire sample should be re-homogenized and subjected to additional particle size reduction. Since not all particles can pass through a sieve, it is also essential to define the minimum acceptable mass percentage that should do so [42]. Although removing larger particles through sieving may improve sample homogeneity, excessive sample loss must be avoided to safeguard the accuracy of quantitative analyses.

Compared to other particle size analysis techniques, sieving is well known for its simplicity and cost-effectiveness. The technique requires relatively low investment in equipment and is straightforward to implement, making it an accessible choice for routine laboratory workflows. Moreover, sieving is considered non-destructive, preserving the chemical composition of the sample and ensuring that the integrity of analytes is maintained for subsequent mycotoxin analysis. In the case of large-scale food testing, sieving provides a means of handling bulk sample volumes. The available standardized protocols further enhance their appeal, promoting consistency and reproducibility across laboratories [43,44]. 

Sieving presents some challenges for food matrices (e.g., peanut butter, dried fruits, fine flours) with high viscosity, low flowability, and a tendency to agglomerate due to high fat, protein, and/or sugar content. Additionally, depending on mesh materials and food matrices, electrostatic charges could also hinder the passage of the particles. Manual sieving of large amounts of samples could be laborious and time-consuming. While mechanical sieving can use agitation, shaking and sonic force, or air flow to facilitate the process, these additions can compromise the simplicity of the technique. Wet sieving could be used to improve flowability, but samples need to be prepared as a slurry first, then dried to determine the mass-based particle size distribution. Sieving results could also be impacted by sample shape and density. For example, the orientation of sample particles with elongated shapes (e.g., fibers) may affect the results, as sieving could measure the second largest dimension of the particle [45,46]. In this case, image analysis may be needed to assess the particle shapes in order to confirm the sieving results. 

While sieving remains a sample preparatory and particle size analysis method for improving homogeneity prior to mycotoxin analysis, its limitations should be carefully evaluated. For food matrices that are not suitable for sieving, complementary techniques such as microscopy, laser diffraction particle size analysis, and/or flow imaging analysis may be used to ensure precise and reliable particle size characterization, thereby enhancing the overall quality of sample homogeneity assessments.

### 2.2. Microscopy

Microscopy functions by magnifying small objects using a combination of lenses and light, or electron beams in the case of electron microscopy, which allows for the visual characterization of particles too small for the naked eye [47]. Microscopy is a valuable tool for particle size analysis, offering unique advantages as well as distinct limitations when evaluating homogeneity based on particle size and size distribution. One of its key strengths lies in its ability to directly visualize particles, allowing researchers to examine not just particle size, but also shape, surface texture, and aggregation. This visual approach enables detailed morphological characterization before and after homogenization—something that statistical techniques like sieving or laser diffraction particle size analysis cannot provide. 

Depending on the microscopy method employed, a broad range of particle sizes (from <1 µm to several millimeters) can be analyzed. Light microscopy, for instance, is relatively low-cost and widely available, making it suitable for routine size assessments. Additionally, microscopy facilitates single-particle analysis, enabling detailed insights into individual particles rather than aggregate measurements alone.

**Figure 2:**
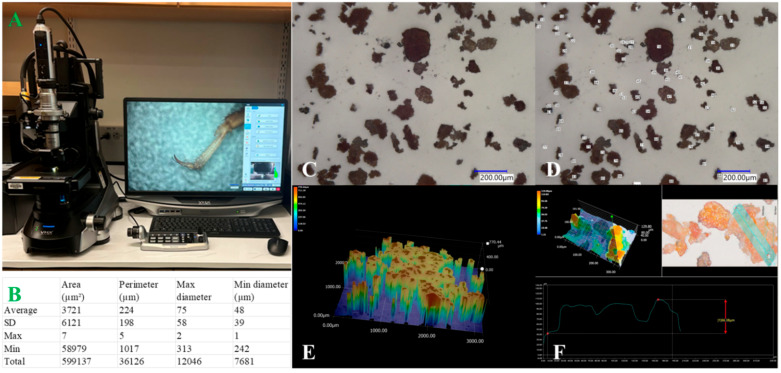
Microscopy-based particle size analysis. (**A**) Digital microscope (Keyence VHX-7000). (**B**) Summary of measured particles (*n* = 161), including area, perimeter, and minimum/maximum diameters. (**C**) Two-dimensional raw image of cocoa bean powder. (**D**) Detected and labeled cocoa bean powder particles. (**E**) Three-dimensional image of cocoa bean powder particles. (**F**) Individual measurement of a single cocoa bean particle. Note: cocoa bean powder is used as a representative case study to demonstrate the workflow of each technique. The tools discussed are applicable across a wide range of food matrices. Details of the microscopy analysis are provided in the Supplementary Materials.

However, microscopy comes with practical challenges. The technique can be time-intensive, often requiring meticulous sample preparation (e.g., particle dispersion), imaging, and, in some cases, manual measurements. The advent of digital microscopy has helped mitigate these issues by introducing automated functions such as autofocusing, image stitching, and 2D/3D scanning. These features allow for the automated acquisition of multiple images as the mechanical stage moves along the X, Y, and/or Z axes, covering a larger field of view. Once captured, particles within these images can be measured automatically using imaging analysis software, partially addressing the limited field of view and manual workflow associated with traditional microscopes. Figure 2 includes an image of a digital microscope (Figure 2A), a summary of particle size analysis (Figure 2B), captured Two–Dimensional (Figure 2C,D) and Three–Dimensional images (Figure 2E), and detailed morphological properties of individual particles (Figure 2F).

The need for well-dispersed samples and limited sample area constrains the number of particles that can be analyzed in a single microscopy measurement. As a result, the statistical robustness of microscopy-based size distribution is generally lower than that of bulk techniques such as sieving, laser diffraction, or flow imaging analysis. In the example shown in Figure 2, only 161 particles were imaged and measured, each exhibiting distinct morphological characteristics (Figure 2B). The combined mass of these particles is less than 0.001 g, compared with a total sample mass of 500 g. This limitation arises from the restricted area of the 1 × 3-inch microscope slide and the need to achieve adequate particle dispersion. Such a small subsample cannot provide representative particle size distribution data for the bulk material, primarily due to insufficient statistical robustness.

Because the error in size distribution is inversely proportional to the square root of the number of particles analyzed, capturing a larger number of particles is essential to reduce error and strengthen statistical validity [47,48]. Achieving this, however, would require repeated sampling and measurement, creating substantial operational challenges in routine testing. Moreover, ensuring consistent dispersion of an adequate number (or mass) of particles across slides is technically difficult and often relies on trial-and-error optimization. Even with multiple measurements, inter- and intra-measurement variability may compromise both statistical reliability and morphological traceability, limiting confidence in the results.

In summary, while microscopy provides valuable morphological information at the individual-particle level, its limited field of view constrains the number of particles that can be analyzed per measurement. This trade-off restricts its capacity to generate statistically robust data for homogeneity assessment. Accordingly, in mycotoxin analysis, microscopy is best positioned as a supplementary tool, offering detailed imaging and morphological insights to complement particle-size-based homogeneity evaluation. Thus, a technique which can provide statistical data for larger sample quantities, such as laser diffraction, is needed.

### 2.3. Laser Diffraction Particle Size Analysis

**Figure 3:**
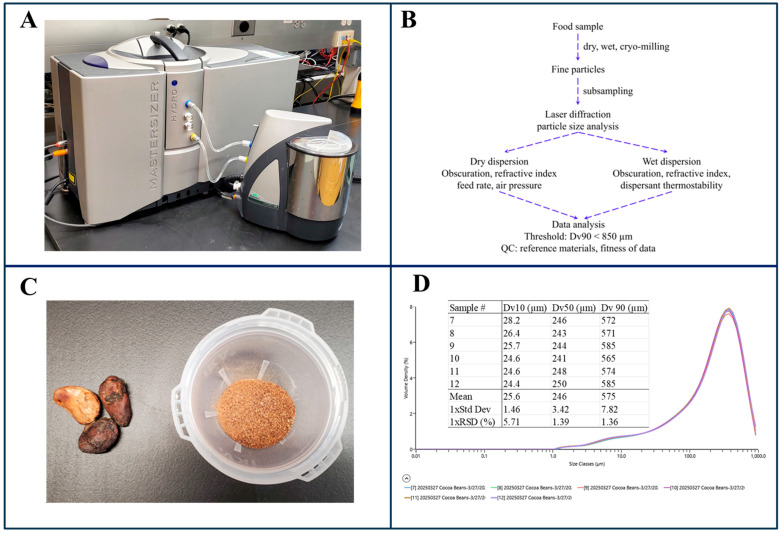
(**A**) Front: Mastersizer 3000 Hydro LV wet dispersion unit; Back: Malvern Mastersizer 3000 laser diffraction particle size analyzer; (**B**) flowchart of laser diffraction particle size analysis; (**C**) left—whole cocoa bean kernels; right—homogenized powder; (**D**) volume-based particle size distribution of the cocoa bean powder (average Dv10, Dv50, and Dv90: 26, 246, and 575 µm). Details of the laser diffraction particle size analysis are provided in the Supplementary Materials.

Laser diffraction particle size analysis estimates particle size based on the light scattering patterns produced by sample particles. Figure 3A shows a picture of a laser diffraction particle size analyzer, including the key hardware profile, a dispersion unit, a measurement cell, and a laser light source. Figure 3B highlights the main steps involved in this technique. First, the sample particles are introduced into the measurement cell via dry (using a gas) or wet dispersion (using a solvent). The laser light source then illuminates the particles, causing the incident light to scatter. Detectors positioned around the measurement cell record the intensity and angular distribution of the scattered light. Using established mathematical models and pre-defined optical properties (e.g., refractive index, obscuration rate), the software algorithm calculates the particle size and size distribution, typically reported as volume-based metrics such as Dv10, Dv50, and Dv90. With a data acquisition rate of up to 10 kHz, laser diffraction particle size analyzers can evaluate a large number of particles within 60–90 s, thereby minimizing sampling error. For example, Figure 3C,D show a homogenized cocoa bean sample where 90% of the total particle volume of the sample consists of particles smaller than 600 µm (Dv90 = 575 µm). 

This technique is supported by well-established mathematical models (e.g., Mie Theory, Fraunhofer Approximation, Rayleigh Scattering Model) [49,50,51], providing a solid theoretical foundation for study design and data interpretation. Additionally, there are established protocols to provide general guidance for routine testing [52,53]. Laser diffraction covers a wide particle size range (e.g., 10–3500 µm) in one measurement [54] and supports both dry and wet dispersion methods, offering flexibility for various food matrices. For dry dispersion, the test portion (the number of particles) is only limited by the availability of the sample. For wet dispersion, the dispersion unit can be customized to accommodate different sample volumes as needed. Unlike sieving, laser diffraction can accommodate samples with high fat and sugar content, provided effective dispersion conditions are established [55]. When paired with an appropriate sampling plan, this technique has potential to enable efficient homogeneity assessment suitable for routine mycotoxin testing [56].

As a volume-based particle sizing technique, laser diffraction can be biased by the presence of a few large particles, since a tenfold increase in particle diameter corresponds to a thousandfold increase in volume. Regardless of the mathematical mode, it assumes a spherical shape of all particles and no multi-scattering. In fact, particle shape can affect measurement accuracy. For example, elongated particles may scatter light differently depending on their orientation, with needle-like particles potentially appearing either as dots or as sticks [57]. Further, accurate analysis depends on optical properties such as refractive index values, which are not always readily available for various food matrices. When applying Mie Theory, these values must be assumed, potentially introducing error. While Fraunhofer Approximation avoids the need for optical properties, it assumes particles are opaque and considers only light scattered at their edges—another simplification that may impact result accuracy [49]. In practical applications, issues like multiple scattering and particle agglomeration due to poor dispersion are not easily identified using laser diffraction particle size analysis alone. To ensure data quality, imaging techniques and reverse data analysis—where the theoretical light scattering pattern derived from experimentally derived particle size distribution is compared with the experimental light scattering pattern—are often necessary [58,59]. For food samples with a wide range of particle sizes and morphological characteristics, a technique such as flow imaging microscopy that integrates the morphological detail of microscopy with the statistical robustness of laser diffraction would provide substantial benefits.

### 2.4. Flow Imaging Microscopy

**Figure 4:**
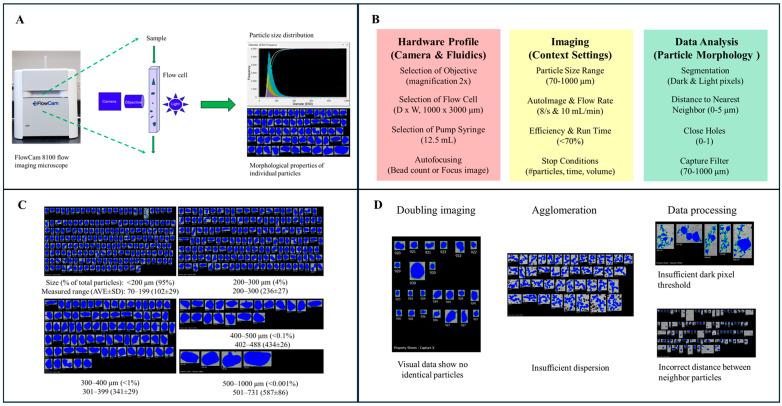
(**A**) Diagram of flow imaging analysis (FlowCam 8100). (**B**) Selection of hardware profile, data acquisition conditions and data processing parameters. (**C**) Particle size distribution pattern and images of measured cocoa bean particles. (**D**) Visual inspection of various troubleshooting issues. Left: no double imaging observed for particles sorted using the same X-Capture value. Middle: agglomeration is identified. Right: imaging data reveal incorrect data processing parameters. Details of the flow imaging analysis are provided in the Supplementary Materials.

Flow imaging microscopy has been extensively applied in fields such as oceanography and the study of protein aggregation [60,61]. A flow imaging microscope, such as the FlowCam 8100 (Figure 4A), integrates an optical system (objectives, lenses), a flow cell, a fluidic pump, a high-speed camera, and a light source. In this system, a sample dispersed in a solvent is pumped through the flow cell, where the high-speed camera captures images of the particles through the optical pathway (comprising the objective lens and other optical components). The accompanying software processes these raw image files to produce two-dimensional representations of individual particles, enabling the measurement of particle size along with other morphological properties (e.g., area, shape, area-based diameter, equivalent spherical diameter, circularity, etc.) and size distribution. Because all detectable particles are imaged and analyzed, flow imaging provides both comprehensive statistical trends for the entire particle population and detailed morphological data at the single-particle level—a valuable combination of features typically offered separately by laser diffraction and microscopy.

To ensure robust and reliable flow imaging analysis, it is essential to carefully select the appropriate hardware settings, optimize the data acquisition efficiency (balancing acquisition rate with the number of particles sampled), and clearly define the parameters for image processing. Figure 4B outlines the critical hardware components and parameters requiring particular attention in the flow imaging workflow. Figure 4C shows the flow imaging analysis of a cocoa bean powder. In total, 30,000 particle images were captured and used to determine the particle size and size distribution. Unlike conventional microscopy, flow imaging functions in a dynamic mode, enabling the analysis of a significantly larger number of particles. This enhances the statistical robustness of particle size distribution assessments. Furthermore, as shown in Figure 4D, the imaging data generated through this technique can be instrumental in identifying and correcting sample preparation issues—such as particle agglomeration or duplicate imaging—and in optimizing image processing parameters. These types of challenges are often difficult to detect using non-imaging particle size analysis techniques like sieving and laser diffraction.

As with traditional microscopy, the field of view in flow imaging is inversely related to the magnification of the objective lens. For example, when employing a 20× objective, the flow cell’s cross-sectional area is substantially smaller than when using a 2× objective. Higher resolution comes at the cost of sampling capacity; achieving finer resolution requires a reduction in flow rate, thereby limiting the number of particles that can be imaged per unit time. As a result, extended measurement times are required to acquire a statistically representative dataset.

Flow imaging also has limitations in terms of the particle size range it can accommodate, which is narrower compared to techniques such as laser diffraction. Without prior knowledge of the sample’s particle size distribution, method development becomes more complex, often requiring the use of multiple objectives and flow cells to capture the full range of particle sizes present. Additionally, flow imaging requires that particles be suspended in a liquid medium. If wet dispersion substantially alters particle morphology, this method may not be appropriate for such samples. 

## 3. Proposed Applications of Existing Particle Size Analysis Techniques

Sound assessment of homogeneity based on particle size relies on analytical tools that offer a balance of precision, efficiency, and detailed morphological insight. The ideal tool should provide high throughput to ensure statistical robustness, cover a wide particle size range—from the sub-micron to the millimeter scale—and capture not just particle size but also shape, surface texture, and aggregation state. In addition to analytical accuracy, practical considerations such as minimal sample preparation, repeatability, automation, and non-destructive analysis are critical for efficient operation. A comprehensive tool should deliver both quantitative metrics (e.g., size distribution statistics) and qualitative morphological data to fully characterize the sample. However, no single method fulfills all these criteria. Each technique offers distinct advantages and inherent limitations, making a complementary approach essential for reliable, in-depth analysis.

Table 1 summarizes the pros and cons of the four particle size analysis techniques discussed. Flow imaging microscopy enables image-based analysis of particles in suspension, offering rich morphological detail. Its use, however, is limited to liquid samples and requires clear suspensions. Laser diffraction is a fast, widely used bulk technique that provides size distribution across a broad range, but it lacks particle shape and individual-particle data. Microscopy allows high-resolution imaging and morphological characterization, but its limited field of view and sample throughput can reduce statistical power. Sieving remains a simple, cost-effective method for analyzing particles in dry powders and food matrices with high flowability. It offers scalability but cannot capture detailed particle shape, size, and distribution. 

By taking advantage of the complementary strengths of different analytical techniques, the mycotoxin research community can apply multiple methods as appropriate to specific matrices and contexts. Such an integrated approach would allow the collection of more comprehensive data, ultimately leading to more reliable particle-size-based homogeneity assessments in mycotoxin analysis.

For instance, methods such as sieving or laser diffraction are well-suited for efficiently characterizing bulk particle size distributions, whereas microscopy or flow imaging microscopy can offer insights into dispersion quality and reveal particle-level anomalies. The optimal combination of techniques depends on factors such as the sample matrix, the particle size range, and the required resolution; nevertheless, a multi-method approach generally enhances both the accuracy and interpretability of the results.

Regardless of the analytical tools employed, evaluating particle-size-based homogeneity should follow a systematic approach. This includes careful consideration of the initial sample mass, the homogenization process, and the procedures used to generate and split subsamples before and after homogenization, as well as the method for selecting test portions [62,63,64]. Additionally, assessing homogeneity based on particle size is more robust when complemented by measurements of within- and between-sample variability in target mycotoxin concentrations. This is especially important given that different mycotoxins may exhibit distinct distribution patterns across various food matrices [56].

## 4. Concluding Remarks and Future Perspectives

Sample homogeneity assessment remains a significant challenge in mycotoxin analysis, particularly for complex and heterogeneous food matrices. Despite available tools and theoretical frameworks, several existing limitations persist across sample preparation, analysis, and quality control.

Gy’s Sampling Theory remains the foundational model for understanding sampling errors, particularly as it relates to particle size. However, its assumptions—such as uniform particle dispersion and random sampling—do not fully reflect the variability and localized “hot spots” of mycotoxin contamination in real food samples [65]. The lack of more nuanced or matrix- and mycotoxin-specific mathematical models hinders precise estimation and control of sampling error in routine testing [66,67].

Sample homogenization is especially challenging given that incoming sample weights can be as high as 27 kg [10], as required by regulatory sampling plans. Processing such large quantities is challenging for most laboratories due to equipment limitations, solvent consumption, waste generation, and cost. Common homogenization methods—dry, wet, or cryogenic milling—each have drawbacks: dry milling may generate heat that degrades analytes; wet milling risks solvent interactions; and cryogenic milling, though effective, is expensive and not suitable for all sample types. There is a pressing need for more efficient, scalable, and non-destructive homogenization tools that can reduce particle size consistently across a wide range of matrices without compromising chemical integrity.

Post-homogenization, tools like riffle splitters and sample dividers are used to generate test portions. However, they can introduce mechanical bias, variability from manual handling, and are often ineffective for fine or cohesive powders. More precise, automated, and standardized subsampling tools are needed to ensure consistency, particularly in high-throughput environments.

Quality control is further constrained by the limited availability of matrix-matched certified reference materials (CRMs), especially for complex food types. Without these, it is difficult to validate homogeneity assessments or confirm method accuracy. Expanding CRM availability is essential for method development, verification, and inter-laboratory comparability. Corn-based mycotoxin-certified reference materials (e.g., NIST SRM 1565 and KRISS CRM 108-01-011) [25,26] are available; however, they are certified solely for the concentrations of selected target mycotoxins. Because particle size data are not certified, these materials cannot serve as CRMs for the quality control of particle size analysis in food matrices.

In summary, advancing sample homogeneity assessment in mycotoxin analysis will require progress on three fronts: developing more nuanced, matrix- and mycotoxin-specific theoretical models to overcome the limitations of Gy’s Sampling Theory; introducing innovative, scalable, and non-destructive technologies for homogenization and automated subsampling to minimize variability and bias; and expanding the availability of matrix-matched certified reference materials to enable reliable method validation, accuracy, and inter-laboratory comparability.

## Figures and Tables

**Table 1:** Comparison of four particle size analysis techniques for evaluating sample homogeneity in mycotoxin analysis. Each technique is assessed based on strengths, limitations, and practical suitability across different food matrices. Throughput and sample volume are relatively ranked using sieving as the benchmark.

Technique	Strengths	Limitations	Best Suited For	Application Range (µm)
Sieving	Large simple size Affordable Non-destructive	Low size resolution Labor/time-intensive Difficulties in agglomerated samples Shape bias	Dry powders with good flowability Large sample volume (e.g., cereal grains, spices)	<850 (No. 20 Sieve) No detailed size distribution
Microscopy	Visual/morphological detail Shape and texture assessment	Low sample throughput Poor statistical power	Supplementary morphological insight Very small sample volume (<0.01 g) (e.g., individual particles)	>1 (Keyence VHX-7000)
Laser Diffraction	Well-established theoretical models Broad size range Fast data acquisition rate	Assumes spherical particles Biased by large particles Estimated optical properties	Routine size distribution across food matrices Small sample volume (~0.5–1.0 g/measurement) (e.g., cereal grains, tree nuts, dried fruits)	10-3500 (Masterszie3000) No morphological information
Flow Imaging Microscopy	Combines morphology with particle size distribution (statistics) Good for dispersion quality control	Limited size range Requires wet dispersion Longer runtime for high size resolution	Well-dispersed samples for both particle size and imaging Small sample volume (~0.05 g/measurement) (e.g., cocoa beans)	2-1000 (FlowCam 8100)

## Data Availability

The original contributions presented in the study are included in the article/Supplementary Material, further inquiries can be directed to the corresponding author.

## Supplementary Materials

The following supporting information can be downloaded at: https://www.mdpi.com/article/10.3390/foods14193294/s1.

## References

[1] Sweeney M.J., Dobson A.D. (1998). Mycotoxin Production by Aspergillus, Fusarium and Penicillium Species. Int. J. Food Microbiol..

[2] Bennett J.W., Klich M. (2003). Mycotoxins. Clin. Microbiol. Rev..

[3] Bullerman L.B., Bianchini A. (2007). Stability of Mycotoxins during Food Processing. Int. J. Food Microbiol..

[4] Lee H.J., Ryu D. (2017). Worldwide Occurrence of Mycotoxins in Cereals and Cereal-Derived Food Products: Public Health Perspectives of Their Co-Occurrence. J. Agric. Food Chem..

[5] Wu F., Groopman J.D., Pestka J.J. (2014). Public health impacts of foodborne mycotoxins. Annu. Rev. Food Sci. Technol..

[6] European Commission Commission Implementing Regulation (EU) 2023/2782 of 14 December 2023 Laying Down the Methods of Sampling and Analysis for the Control of the Levels of Mycotoxins in Food and Repealing Regulation (EC) No 401/2006. https://eur-lex.europa.eu/eli/reg_impl/2023/2782/oj.

[7] U.S. Food and Drug Administration (FDA) (2024). Compliance Program Guidance Manual. Chapter 07—Molecular Biology and Natural Toxins. https://www.fda.gov/media/140749/download?attachment.

[8] Health Canada Food Safety—Chemical Contaminants—Natural Toxins. https://www.canada.ca/en/health-canada/services/food-nutrition/food-safety/chemical-contaminants/natural-toxins.html.

[9] Whitaker T.B. (2006). Sampling Foods for Mycotoxins. Food Addit. Contam..

[10] U.S. Food and Drug Administration (FDA) 2025 Investigations Operations Manual (IOM), Chapter 4: Sampling. https://www.fda.gov/inspections-compliance-enforcement-and-criminal-investigations/inspection-references/investigations-operations-manual.

[11] Food and Agriculture Organization (FAO) (2025). Mycotoxin Sampling Tools. https://tools.fstools.org/mycotoxins/.

[12] Shotwell O.L., Goulden M.L., Botast R.J., Hesseltine C.W. (1975). Mycotoxins in hot spots in grains. 1. Aflatoxin and zearalenone occurrence in stored corn. Cereal Chem..

[13] Maestroni B., Cannavan A., De Saeger S. (2011). Sampling strategies to control mycotoxins. Determining Mycotoxins and Mycotoxigenic Fungi in Food and Feed.

[14] U.S. Department of Agriculture (USDA) Grain Fungal Diseases and Mycotoxin Reference. https://www.ams.usda.gov/sites/default/files/media/FungalDiseaseandMycotoxinReference2017.pdf.

[15] AOAC (2023). Natural Toxins. Official Methods of Analysis.

[16] Biselli S., Persin C., Syben M. (2008). Investigation of the Distribution of Deoxynivalenol and Ochratoxin A Contamination within a 26 t Truckload of Wheat Kernels. Mycotoxin Res..

[17] Qi Z., Tian L., Zhang H., Lei Y., Tang F. (2023). Fungal Community Analysis of Hot Spots in Bulk Maize under Different Storage Conditions. LWT.

[18] Betina V. (1985). Thin-Layer Chromatography of Mycotoxins. J. Chromatogr..

[19] Greer B., Chevallier O., Quinn B., Botana L.M., Elliott C.T. (2021). Redefining Dilute and Shoot: The Evolution of the Technique and Its Application in the Analysis of Foods and Biological Matrices by Liquid Chromatography Mass Spectrometry. TrAC Trends Anal. Chem..

[20] Malachova A., Krska R., Berthiller F., Sulyok M., Beltrán E. (2015). Multi-Toxin Determination in Food—The Power of “Dilute and Shoot” Approaches in LC–MS–MS. LCGC Eur..

[21] Zhang K., Schaab M.R., Southwood G., Tor E.R., Aston L.S., Song W., Eitzer B., Majumdar S., Lapainis T., Mai H. (2017). A Collaborative Study: Determination of Mycotoxins in Corn, Peanut Butter, and Wheat Flour Using Stable Isotope Dilution Assay (SIDA) and Liquid Chromatography-Tandem Mass Spectrometry (LC-MS/MS). J. Agric. Food Chem..

[22] Sulyok M., Stadler D., Steiner D., Krska R. (2020). Validation of an LC-MS/MS-based dilute-and-shoot approach for the quantification of > 500 mycotoxins and other secondary metabolites in food crops: Challenges and solutions. Anal. Bioanal. Chem..

[23] Pitard F.F. (1989). Pierre Gy’s Sampling Theory and Sampling Practice.

[24] Gerlach R.W., Nocerino J.M. (2003). EPA/600/R-03/027 Guidance for Obtaining Representative Laboratory Analytical Subsamples from Particulate Laboratory Samples.

[25] Phillips M.M., Seal T.M.L., Ness J.M., Zhang K. (2019). Development and Characterization of a Multimycotoxin Reference Material. J. AOAC Int..

[26] Gab-Allah M.A., Getachew Lijalem Y., Yu H., Lee S., Baek S.Y., Han J., Choi K., Kim B. (2023). Development of a Certified Reference Material for the Accurate Determination of Type B Trichothecenes in Corn. Food Chem..

[27] Pinelo M., Tress A.G., Pedersen M., Arnous A., Meyer A.S. (2007). Effect of Cellulases, Solvent Type and Particle Size Distribution on the Extraction of Chlorogenic Acid and Other Phenols from Spent Coffee Grounds. Am. J. Food Technol..

[28] Brewer L.R., Kubola J., Siriamornpun S., Herald T.J., Shi Y.C. (2014). Wheat Bran Particle Size Influence on Phytochemical Extractability and Antioxidant Properties. Food Chem..

[29] Damiani T., Righetti L., Suman M., Galaverna G., Dall’Asta C. (2019). Analytical issue related to fumonisins: A matter of sample comminution?. Food Control..

[30] Zhang Y., Wu Q.-K., Han Y.-T., Jiang Y., Xie G. (2023). Effect of Grinded Particle Size on the Determination of Aflatoxin B1 in Maize Evaluated by Fractional Preparation Method. Sci. Technol. Cereals Oils Foods.

[31] Spanjer M.C., Scholten J.M., Kastrup S., Jörissen U., Schatzki T.F., Toyofuku N. (2006). Sample comminution for mycotoxin analysis: Dry milling or slurry mixing?. Food Addit. Contam..

[32] Bircan C. (2009). Comparison of homogenization techniques and incidence of aflatoxin contamination in dried figs for export. Food Addit. Contam..

[33] Lippolis V., Pascale M., Valenzano S., Visconti A. (2012). Comparison of slurry mixing and dry milling in laboratory sample preparation for determination of ochratoxin A and deoxynivalenol in wheat. J. AOAC Int..

[34] Tittlemier S.A., Cramer B., DeRosa M.C., Dzuman Z., Kodikara C., Malone R., Maragos C., Suman M., Sumarah M.W. (2025). Developments in Analytical Techniques for Mycotoxin Determination: An Update for 2023–24. World Mycotoxin J..

[35] (2024). Reference Materials—Approaches for Characterization and Assessment of Homogeneity and Stability.

[36] (2003). Foodstuffs—Determination of Aflatoxin B1, and the Total Content of Aflatoxins B1, B2, G1 and G2 in Cereals, Nuts and Derived Products—High-Performance Liquid Chromatographic Method.

[37] (2007). (Main) Foodstuffs—Determination of Aflatoxin B1 and the sum of Aflatoxin B1, B2, G1 and G2 in Hazelnuts, Peanuts, Pistachios, Figs, and Paprika Powder—High Performance Liquid Chromatographic Method with Post-Column Derivatisation and Immunoaffinity Column Cleanup.

[38] Council for Agricultural Science and Technology (CAST) (1989). Mycotoxins. Economic and Health Risks. Council for Agricultural Science and Technology Task Force Report. No. 116. Ames, Iowa. https://cast-science.org/publication/mycotoxins-economic-and-health-risks/.

[39] Council for Agriculture Science and Technology (CAST) (2003). Mycotoxins: Risks in Plant, Animal, and Human Systems. Task Force Report 139. Ames, Iowa. https://cast-science.org/publication/mycotoxins-risks-in-plant-animal-and-human-systems/.

[40] Schatzki T.F., Toyofuku N. (2003). Sample Preparation and Presampling of Pistachios. J. Agric. Food Chem..

[41] Sasser M., Herrman T.J., Lee K.M. (2018). Evaluation of Coregulation as a Governance Option to Manage Aflatoxin Risk in Texas Maize. J. Food Prot..

[42] Kos G., Lohninger H., Mizaikoff B., Krska R. (2007). Optimisation of a sample preparation procedure for the screening of fungal infection and assessment of deoxynivalenol content in maize using mid-infrared attenuated total reflection spectroscopy. Food Addit. Contam..

[43] (2023). USP <786> Particle Size Distribution Estimation by Analytical Sieving. https://www.usp.org/harmonization-standards/pdg/general-chapters/analytical-sieving.

[44] (2013). Test Sieves—Technical Requirements and Testing.

[45] Jillavenkatesa A., Dapkunas S.J., Lum L.-S.H. (2001). Particle Size Characterization. NIST Special Publication 960–1. https://nvlpubs.nist.gov/nistpubs/Legacy/SP/nistspecialpublication960-1.pdf.

[46] Bartley P.C., Jackson B.E., Fonteno W.C. (2019). Effect of particle length to width ratio on sieving accuracy and precision. Powder Technol..

[47] Murphy D.B., Davidson M.W. (2012). Fundamentals of Light Microscopy and Electronic Imaging.

[48] Masuda H., Linoya K. (1971). Theoretical study of the scatter of experimental data due to particle size distribution. J. Chem. Eng. Jpn..

[49] De Boer G.B.J., de Weerd C., Thoenes D., Goossens H.W.J. (1987). Laser Diffraction Spectrometry: Fraunhofer Diffraction Versus Mie Scattering. Part. Part. Syst. Charact..

[50] Jones A.R. (1999). Light scattering for particle characterization. Prog. Energy Combust. Sci..

[51] Wriedt T., Hergert W., Wriedt T. (2012). Mie Theory: A Review. The Mie Theory.

[52] (2024). Laser Diffraction Measurements—Good Practice.

[53] (2020). Particle Size Analysis—Laser Diffraction Methods.

[54] Malvern Panalytical Mastersizer 3000. https://www.malvernpanalytical.com/en/assets/mastersizer%203000%20brochure%20(en)_tcm50-58994.pdf.

[55] Zhang K., Tan S., Xu D. (2022). Determination of Mycotoxins in Dried Fruits Using LC-MS/MS—A Sample Homogeneity, Troubleshooting and Confirmation of Identity Study. Foods.

[56] Zhang K., Tran I., Tan S. (2023). Characterization of Particle-Size-Based Homogeneity and Mycotoxin Distribution Using Laser Diffraction Particle Size Analysis. Toxins.

[57] Agimelen O.S., Mulholland A.J., Sefcik J. (2017). Modelling of artefacts in estimations of particle size of needle-like particles from laser diffraction measurements. Chem. Eng. Sci..

[58] Agrawal Y.C., McCave I.N., Riley J.B., Syvitski J.P.M. (1991). Laser diffraction size analysis. Principles, Methods and Applications of Particle Size Analysis.

[59] Blott S.J., Pye K. (2006). Particle size distribution analysis of sand-sized particles by laser diffraction: An experimental investigation of instrument sensitivity and the effects of particle shape. Sedimentology.

[60] Sieracki C.K., Sieracki M.E., Yentsch C.S. (1998). An Imaging-in-Flow System for Automated Analysis of Marine Microplankton. Mar. Ecol. Prog. Ser..

[61] Zölls S., Weinbuch D., Wiggenhorn M., Winter G., Friess W., Jiskoot W., Hawe A. (2013). Flow Imaging Microscopy for Protein Particle Analysis—A Comparative Evaluation of Four Different Analytical Instruments. AAPS J..

[62] (2007). Particulate Materials—Sampling and Sample Splitting for the Determination of Particulate Properties.

[63] Gerlach R.W., Dobb D.E., Raab G.A., Nocerino J.M. (2002). Gy Sampling Theory in Environmental Studies. 1. Assessing Soil Splitting Protocols. J. Chemom..

[64] Gerlach R.W., Nocerino J.M., Ramsey C.A., Venner B.C. (2003). Gy Sampling Theory in Environmental Studies: 2. Subsampling Error Estimates. Anal. Chim. Acta.

[65] Chavez R.A., Xianbin Cheng X., Herrman T.J., Stasiewicz M.J. (2022). Single kernel aflatoxin and fumonisin contamination distribution and spectral classification in commercial corn. Food Control.

[66] Casado M.R., Parsons D.J., Weightman R.M., Magan N., Origgi S. (2009). Geostatistical analysis of the spatial distribution of mycotoxin concentration in bulk cereals. Food Addit. Contam. Part A.

[67] Casado M.R., Parsons D.J., Weightman R.M., Magan N., Origgi S. (2009). Modelling a two-dimensional spatial distribution of mycotoxin concentration in bulk commodities to design effective and efficient sample selection strategies. Food Addit. Contam. Part A.

