# Supplementary material for: Opinion Piece: Tools for Particle-Size-Based Homogeneity Assessments in Mycotoxin Analysis
Source: Foods. 2025 Sep 23;14(19):3294. doi: 10.3390/foods14193294 (PMC12523435; doi:10.3390/foods14193294)
Supplement: Supplementary file 1 [file foods-14-03294-s001.zip › foods-3849049-supplementary.pdf]

Opinion

Opinion Piece: Tools for Particle Size-Based Homogeneity Assessments in Mycotoxin Analysis

Kai Zhang[1,*] and Grace Reichard[1]

[1]Food and Drug Administration, Human Foods Program, Office of Chemistry and Toxicology 5001 Campus Drive, College Park, MD 20740, USA
* Correspondence: Email: kai.zhang@fda.hhs.gov; Tel: +1-240-402-2318

Supporting information

Opinion Piece: Particle Size-Based Homogeneity Assessment Tools for Mycotoxin Analysis
Kai Zhang [1,*], Grace Reichard [1]
[1]Food and Drug Administration, Human Foods Program, Office of Chemistry and Toxicology
5001 Campus Drive, College Park, MD 20740
*Correspondence: Email: kai.zhang@fda.hhs.gov; Tel: 240-402-2318

Materials and Methods
Sample preparation
The whole cocoa bean kernel sample used for this study was a generous gift from Dr. Badrul Hisyam Zainudin at Malaysian Cocoa Board. Whole cocoa bean kernels with shells (500 g) were put in a freezer (-80 ^{o}C) overnight and blended using a Blixer 4 blender for 3 min (Robot Coupe, Inc., Ridgeland, MS, USA). Then a subsample (25 g) was further homogenized using an IKA mill (IKA Inc., Wilmington, NC, USA) at 25,000 rpm for 1.5 min prior to particle size analysis.

Laser diffraction particle size analysis
Following a previously developed particle size analysis protocol [57], cocoa bean powder was introduced into a Mastersizer 3000 (Malvern Panalytical Inc., Malvern, UK) using a wet dispersion unit. Methanol was used as the dispersant. Briefly, samples were gradually introduced into a Hydro LV Disperser (Malvern Panalytical Inc.). To evenly suspend and disperse the sample particles in methanol, the stirring rate of the disperser was set at 3500 rpm, minimizing agglomeration. When the obscuration reading reached between 20% and 30%, the measurement was initiated and there were six replicates for each measurement. The dispersed particles were also used for microscopy analysis and flow image analysis.

Microscopy analysis
Static image analysis of the cocoa bean particles was performed using a digital microscope (Keyence Corp. of America, Itasca, IL, USA). A small drop of the suspension was pipetted from the Hydro LV Disperser onto a clean microscope slide (1″×3″) (Fisherbrand® 2R disposable microscope slides, Thermo Fisher Scientific, Waltham, MA, USA) and a coverslip was added to flatten the sample layer. A 100x objective was selected to cover a particle size range of 1-1000 µm. Autofocusing, image stitching, and depth composition features were used to capture 2D and 3D images of the particles on the slide. Particle size and morphological properties were measured using VHX software (Keyence Corp. of America, Itasca, IL, USA).

Flow imaging analysis

A FlowCam 8100 system (Yokogawa Fluid Imaging Technologies, Scarborough, ME, USA) was used to perform flow imaging analysis. As cocoa bean powder can only be introduced into the FlowCam and pumped through the flow cell via a fluidic fashion, 30 mL of the dispersed sample was pipetted out from the Hydro LV Disperser and loaded on the sample reservoir of the 8100 FlowCam, followed by initiating the fluidic pump. On the FlowCam imaging analyzer, a 2x objective, FOV1000 flow cell (1000 µm × 3000 µm, depth × width) and 12.5 mL syringe pump were installed to collect raw image data for particle sizes ranging from 70-1000 µm. The image output was 8-bit grayscale TIFF. The VisualSpreadsheet software (version 6.0, Yokogawa Fluid Imaging Technologies, Scarborough, ME, USA) was used to operate the instrument and process the raw image data files. Prior to sample analysis, the system was calibrated using the autofocusing feature and Duke Standards 4K100, NIST traceable polymer microspheres (Thermo Fisher Scientific, Waltham, MA, USA). The sample volume was set at 30 mL and the flow rate was set at 3 mL/min with a stop condition when the particle count reached 30,000. The efficiency was 66.6% with an automate rate at 3 frames per second. Raw images (1000) were saved so that different processing parameters (e.g., distance to nearest neighbor, intensity threshold for both dark and light pixel, close holes, and capture filter) can be used to reprocess for comparison.
